# A Setmelanotide-like Effect at MC4R Is Achieved by MC4R Dimer Separation

**DOI:** 10.3390/biom12081119

**Published:** 2022-08-15

**Authors:** Nanina Reininghaus, Sarah Paisdzior, Friederike Höpfner, Sabine Jyrch, Cigdem Cetindag, Patrick Scheerer, Peter Kühnen, Heike Biebermann

**Affiliations:** 1 Charité—Universitätsmedizin Berlin, Corporate Member of Freie Universität Berlin and Humboldt-Universität zu Berlin, Institute of Experimental Pediatric Endocrinology, Augustenburger Platz 1, 13353 Berlin, Germany; 2 Charité—Universitätsmedizin Berlin, Corporate Member of Freie Universität Berlin and Humboldt-Universität zu Berlin, Group Protein X-ray Crystallography and Signal Transduction, Institute of Medical Physics and Biophysics, 10117 Berlin, Germany; 3 DZHK (German Centre for Cardiovascular Research), Partner Site Berlin, 13353 Berlin, Germany

**Keywords:** melanocortin 4 receptor, MC4R, obesity, homodimer, G_q/11_, signaling, GPCR

## Abstract

Melanocortin 4 receptor (MC4R) is part of the leptin-melanocortin pathway and plays an essential role in mediating energy homeostasis. Mutations in the *MC4R* are the most frequent monogenic cause for obesity. Due to increasing numbers of people with excess body weight, the MC4R has become a target of interest in the search of treatment options. We have previously reported that the MC4R forms homodimers, affecting receptor G_s_ signaling properties. Recent studies introducing setmelanotide, a novel synthetic MC4R agonist, suggest a predominant role of the G_q/11_ pathway regarding weight regulation. In this study, we analyzed effects of inhibiting homodimerization on G_q/11_ signaling using previously reported MC4R/CB1R chimeras. NanoBRET^TM^ studies to determine protein–protein interaction were conducted, confirming decreased homodimerization capacities of chimeric receptors in HEK293 cells. G_q/11_ signaling of chimeric receptors was analyzed using luciferase-based reporter gene (NFAT) assays. Results demonstrate an improvement of alpha-MSH-induced NFAT signaling of chimeras, reaching the level of setmelanotide signaling at wild-type MC4R (MC4R-WT). In summary, our study shows that inhibiting homodimerization has a setmelanotide-like effect on G_q/11_ signaling, with chimeric receptors presenting increased potency compared to MC4R-WT. These findings indicate the potential of inhibiting MC4R homodimerization as a therapeutic target to treat obesity.

## 1. Introduction

The prevalence of obesity has increased immensely over the past years, not only for the adult population but also among children and adolescents [1,2]. Obesity poses a worldwide health challenge as it is a major risk factor for developing conditions such as diabetes type 2 [3] and cardiovascular diseases [4]. This reality highlights the importance of finding treatment options to target this strain on global health. Bariatric surgery has strong evidence of efficacy and is especially recommended for specific patients with severe obesity [5]. Surgery is, however, not an option for all patients suffering from obesity, especially not for children with weaknesses in the leptin–melanocortin pathway [6,7,8,9]. The leptin–melanocortin signaling pathway regulates energy homeostasis in the hypothalamus, therefore playing a key role in regulating body weight [10]. Recently, we reported setmelanotide as effective in treating patients with proopiomelanocortin (*POMC)* or leptin receptor (*LEPR*) deficiency [11,12]. Only a small subset of patients profit from this medical intervention. Therefore, an option for a broader group of patients is urgently needed. 

The melanocortin-4 receptor (MC4R) is one of the key G-protein-coupled receptors (GPCR) in the leptin–melanocortin signaling pathway. Mutations in the *MC4R* account for up to 5% of obesity cases and are the most frequent monogenic cause for obesity [13,14,15]. Due to the discovery that GPCRs can act as dimers, thus affecting signaling abilities, finding ways to study dimerization behavior and respective effects has become a common field of interest [16,17,18]. Although several instances of MC4R homo- and heterodimerization have been reported over the years [19], the MC4R does not interact with the phylogenetically close cannabinoid receptor type 1 (CB1R) [19,20]. By using MC4R/CB1R chimeras, we were able to recently show that MC4R homodimer separation leads to an increase in G_s_ activation in COS-7 cells [21]. Activation of G_s_ signaling is the major signaling pathway of MC4R [22], and the 3D structures of various MC4R/G_s_ complexes have recently been solved, including by us [23,24]. However over recent years, it was identified that other pathways are activated as well [25]. In particular, G_q/11_ signaling seems to play a so far underestimated role in MC4R-induced weight regulation [12,26].

Therefore, the objective of this study was to investigate if homodimer separation of the MC4R also has an effect on G _q/11_ activation. For this purpose, reported MC4R/CB1R chimeras were investigated using human cell line HEK293 cells, specifically focusing on G _q/11_ activation.

## 2. Materials and Methods

### 2.1. Ligands and Plasmids

The endogenous ligand alpha-MSH was purchased from Merck (Taufkirchen, Germany). MC4R-WT, CB1R cDNA was amplified from genomic DNA and cloned into eukaryotic expression vector pcDps. MC4R-H158R as well as MC4R/CB1R chimeric receptors were recently generated in this expression vector [21]. For protein–protein interaction NanoBRET™ assays, the cDNA of MC4R, CB1R, and H158R as well as chimeric constructs 1-7 were cloned into Flexi expression vectors, respectively, pFC14A (Promega, Mannheim, Germany) and pFC32K (Promega, Mannheim, Germany), according to the manufacturer’s protocol.

### 2.2. Cell Culture

Human embryonic kidney 293 (HEK293) cell line was purchased from ATCC and regularly tested for mycoplasma contamination using DAPI staining. Cells were maintained in minimal essential medium (MEM, Gibco, Waltham, MA, USA) supplemented with 5% fetal bovine serum (FBS, Gibco, Waltham, MA, USA) and NEAA incubated at 37 °C in humidified air containing 5% CO_2_. For the BRET assays, 4 × 10^5^ HEK293 cells per well were seeded in 6-well plates for transfection. For the donor saturation assays (DSA), 4 × 10^5^ HEK293 cells per well were seeded in 12-well plates. For GloSensor™ assays, 1.5 × 10^4^ cells per well were seeded in white 96-well plates (Corning, Costar, AZ, USA). For the NFAT luciferase-based reporter gene assay, 1.5 × 10^4^ cells per well were seeded in poly-L-lysine-coated (Gibco, Waltham, MA, USA) 96-well translucent plates (Falcon, Kaiserslautern, Germany) and incubated for 24 h. 

### 2.3. Transfection

For BRET assays, HEK293 cells were transfected 4–6 h after seeding. Transfection was performed using 8 µL FuGene HD (Promega, Mannheim, Germany) and 1.4 µg DNA in Opti-MEM (Gibco, Waltham, MA, USA). The BRET partners were co-transfected using a NL/HT ratio of 1:5 (200 ng: 1 µg). In addition, 200 ng Carrier DNA (pGEM3Z) was added. For GloSensor™, HEK293 cells were transfected 24 h after seeding with 60 ng plasmid DNA and 0.6 µL Metafectene (Biontex, Munich, Germany), according to the manufacturer’s protocol. An additional 60 ng of pGloSensor™-22F cAMP Plasmid was added. For reporter gene assays, HEK293 cells were transfected 24 h after seeding with 45 ng plasmid DNA and 0.45 µL Metafectene (Biontex, Munich, Germany), according to the manufacturer’s protocol. A total of 45 ng of reporter DNA (pGL4.3(luc2P/NFAT/Hygro)) was additionally transfected per well in MEM without supplements. 

### 2.4. Determination of Protein–Protein Interaction via NanoBRET™

The interaction between MC4R-WT, CB1R, and the chimeric receptors was determined using NanoBRET™ (Promega, Mannheim, Germany). The receptors were either C-terminally tagged with HaloTag (HT) as an energy acceptor or C-terminally fused with NanoLuc (NL) as an energy donor. Then, 20 h after transfection, cells were detached from the wells and centrifuged at 130× *g* for 5 min and the pellet was resuspended in Opti-MEM (Gibco) without phenol red, supplemented with 4% FBS (Gibco). Cells were adjusted to 2 × 10^5^ cells/mL and divided into two pools, adding either DMSO or HaloTag® Ligand 618. The cells were then reseeded into white opaque 96-well plates (Corning, Costar) and incubated for 4–6 h at 37° and 5% CO_2_. Measurements were conducted using the Berthold Mithras LB 940, injecting 25 µL/well of NanoGlo® substrate and measuring donor and acceptor emission at 460 nm and 618 nm, respectively. The BRET Ratio was calculated by dividing acceptor emission by donor emission. In order to correct for background bleedthrough, the background ratio (no acceptor DMSO control) was subtracted, and the unit was changed into miliBRET (mBU).

(1) $$\mathrm{BRET}\;\mathrm{Ratio}=\frac{\mathrm{emission}\;\mathrm{acceptor}\;\left(618\;\mathrm{nm}\right)}{\mathrm{emission}\;\mathrm{donor}\;\left(460\;\mathrm{nm}\right)}$$

(2) $$\mathrm{BRET}\;\mathrm{Ratio}=\;(\mathrm{BRET}\;{\mathrm{Ratio}}_{\mathrm{sample}}-\mathrm{BRET}{\mathrm{Ratio}}_{\mathrm{no}\;\mathrm{acceptor}\;\mathrm{control}})\times 1000$$

For graphical depiction the delta BRET was calculated

(3) $$\Delta \mathrm{BRET}=\mathrm{BRET}{\mathrm{Ratio}}_{\mathrm{chimeric}\;\mathrm{receptor}}-\mathrm{BRET}{\mathrm{Ratio}}_{\mathrm{WT}-\mathrm{MC}4R}$$

### 2.5. Determination of cAMP Accumulation via GloSensor^TM^

G_s_ signaling was measured using cAMP assay GloSensor™, enabling real-time measurements of cAMP accumulation. HEK293 cells were transfected with either MC4R-WT or chimeric receptors. Two days after transfection, cells were equilibrated with a mixture of 88% CO_2_-dependent medium (Gibco, Waltham, MA, USA), 10% FCS, and 2% GloSensor™ cAMP Reagent. In case of PTX pretreatment, 10 µL of 50 ng/mL PTX was added to cells 18 h before stimulation. Non-PTX-treated cells received 10 µL MEM without supplements (Gibco Minimum Essential Media, Waltham, MA, USA) per well at the same time point. Bioluminescence was quantified using a Berthold Microplate Reader (Mithras LB940, Berthold Technologies GmbH and Co., Bad Wildbad, Germany). After basal measurement for 10 min, cells were stimulated with 1 µM alpha-MSH, or 1 µM setmelanotide, and measured for 21 times at 2 min intervals. Cells stimulated with 1 µM isoproterenol served as an internal control. GloSensor™ results were expressed as relative luminescence units (RLU). The total cAMP formation was assessed in the time–response curve by calculating the area under the curve (AUC).

### 2.6. Measurements of PLC Aactivation Using Reporter Gene Assays

In order to determine phospholipase C (PLC) activation, luciferase-based reporter gene assays were conducted. Then, 48 h after transfection, cells were challenged with alpha-MSH or setmelanotide, using decreasing concentrations (10^−5^ M to 10^−10^ M) for 6 h at 37 °C and 5% CO_2_. In a case of PTX pretreatment experiments, 50 ng/mL PTX was added to cells 18 h before stimulation. After incubation, stimulation was stopped by discarding the media, and subsequent cell lysis was induced by adding 50 µL passive lysis buffer (PLB; Promega, Mannheim, Germany). For measurements, 10 µL of the lysate was transferred to a white opaque 96-well plate. Measurements were conducted by injecting 40 µL firefly luciferase substrate (Promega, Mannheim, Germany), and luminescence was determined with a plate reader (Mithras LB940). Signaling bias was analyzed using the formula described by Kenakin [27]:

(4) $$\Delta \log\left(\frac{E_{\max}}{{\mathrm{EC}}_{50}}\right)=\log\left(\frac{E_{\max,\;A}}{{\mathrm{EC}}_{50,A}}\right)-\log\left(\frac{E_{\max,\;B}}{{\mathrm{EC}}_{50,\;B}}\right)$$

with A referring to the respective chimeric receptor and B being the reference (MC4R-WT). E_max_ was determined from concentration–response curves and depicts the maximal response (efficacy); EC_50_ describes the potency. Bias was further calculated, creating the antilog.

(5) $$\mathrm{bias}=10^{\Delta \log\left(\frac{E_{\max}}{{\mathrm{EC}}_{50}}\right)}$$

### 2.7. Statistical Analysis

All data represent mean ± SEM. Statistical testing and calculation of area under the curve were performed using GraphPad Prism 9.3.1 software (San Diego, CA, USA). For concentration–response curves, a non-linear regression model for sigmoidal response was used. Hyperbolic curves were adapted by the non-linear regression of standard curves to interpolate. Significance between parameters was either calculated using a one-way ANOVA with Kruskal–Wallis test or the Mann–Whitney test, with *p* ≤ 0.05 set as significant. For more details, see descriptions in the figures below.

## 3. Results

**Figure 1:**
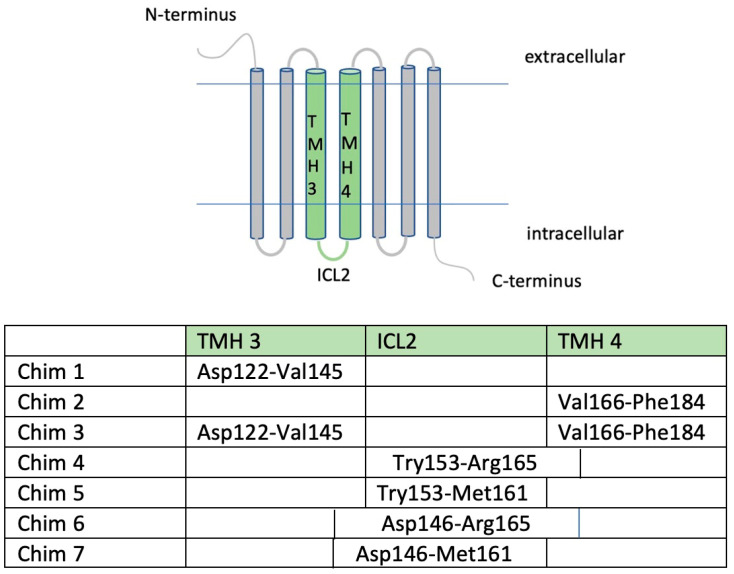
**Arrangement of MC4R/CB1R chimeric constructs.** The table illustrates amino acid sequences and regions interchanged with the CB1R via overlap PCR. Chimera specification: Chim 1: substitution of TMH3; Chim 2: substitution of TMH4; Chim 3: substitution of TMH3 and TMH4 regions; Chim 4: substitution of ICL2 and intracellular parts of TMH4; Chim 5: subsitution of ICL2; Chim 6: substitution of intracellular parts of TMH3, ICL2, and intracellular parts of TMH4; Chim 7: substitution of intracellular parts of TMH3 and ICL2 [21]. Cloning of HaloTag (HT) and NanoLuc (NL) was performed using Sgfl and EcoICRI enzymes. The HT Tag is situated at the C-terminal end of the gene after a HT7 linker and a TEV protease recognition sequence. The NL is also situated at the C-terminal, NL protein coding region following after a Linker (region 14471461). The vectors were obtained from Promega, and cloning was conducted according to Flexi^®^ Vector Systems protocol.

The aim of this study was to investigate MC4R oligomeric behavior and downstream signaling in a human cell model. Therefore, we worked with HEK293 cells, a well-established cell model for working with GPCRs. We recently reported MC4R monomerization in COS-7 cells using an ELISA approach [21]. For this study, the reported chimeric constructs (Figure 1) were used for application of a NanoBRET™ assay studying protein–protein interaction, and therefore appropriated tags were added.

### 3.1. Homodimerization Capacities of Chimeric CB1R/MC4R and MC4R-H158R Mutation Were Reduced when Compared to MC4R-WT

**Figure 2:**
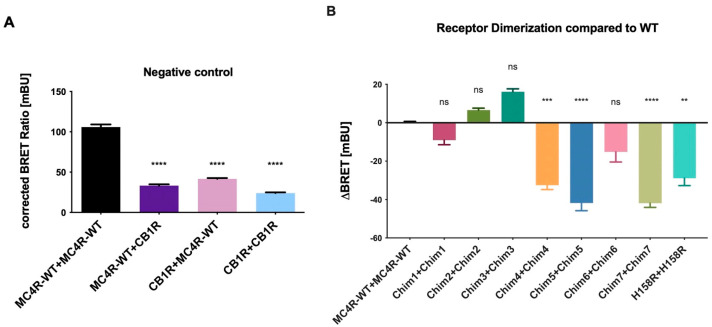
**Receptor dimerization of MC4R chimeras compared to MC4R-WT.** Dimerization of chimeric MC4R/CB1R receptors and H158R gain of function mutation was analyzed by performing NanoBRET™ assays. HEK293 cells were co-transfected with either BRET partners, C-terminally tagged with the energy donor NanoLuc or the protein tag HaloTag, able to bind the energy acceptor, the NanoBRET^TM^ ligand 618. (**A**) CB1R is a non-interactive partner of MC4R and served as the negative control. Data are shown as BRET ratio in milliBRET units (mBU). (**B**) ΔBRET values were calculated as difference between BRET ratios of chimeras and MC4R-WT. Negative values represent a decrease in dimerization capacities compared to MC4R-WT dimerization. Data represent three independent experiments, each performed in triplicate. Values represent mean ± SEM of calculated BRET ratios. A one-way ANOVA with Kruskal–Wallis test was performed for statistical analysis, and the mean of the WT column was compared to the mean of all the other columns. Statistical significance was defined as ** *p* < 0.01, *** *p* < 0.001, and **** *p* < 0.0001.

Heterodimerization of MC4R-WT and CB1R as negative control was tested, pairing BRET partners in two possible ways, assessing the interaction between MC4R tagged with NL plus CB1R tagged with HT and an interaction of the pairing CB1R NL plus MC4R HT. We found receptor heterodimerization of MC4R-WT and CB1R was significantly reduced for both groupings when compared to MC4R-WT homodimerization as expected. CB1R homodimerization was also decreased compared to MC4R-WT homodimerization (Figure 2A). The NanoBRET™ assay was therefore chosen as a suitable method to investigate receptor dimerization of chimeric MC4R/CB1R variants. Additionally, the MC4R H158R, a classified gain-of-function mutation located in the second intracellular loop (ICL2) [25], which has decreased ability to form homodimers, was included. The MC4R-CB1R chimeras Chim 1, Chim 2, and Chim 3 showed no significant (Chim 1) or increased (Chim 2, Chim 3) receptor–receptor interaction, compared to MC4R-WT. Due to these results and the fact that Chim 1, Chim 2, and Chim 3 have shown impaired cell surface expression in previous experiments conducted in COS-7 cells, they were excluded from further experiments [21]. Chim 4, Chim 5, Chim 7, and the H158R gain-of function mutation showed significantly impaired dimerization capacities. Chim 5 and Chim 7 exhibited the maximum suppression of dimer formation (Figure 2B). The results are in line with previous results deriving from sandwich ELISA experiments conducted in COS-7 cells [21]. Donor saturation assays (DSA) were conducted for WT, Chim 7, and H158R to test the specificity of homodimer interaction. A specific interaction could be verified for all three constructs (Supplementary Information, Figure S1). 

### 3.2. Effect of Homodimerization on cAMP Formation

**Figure 3:**
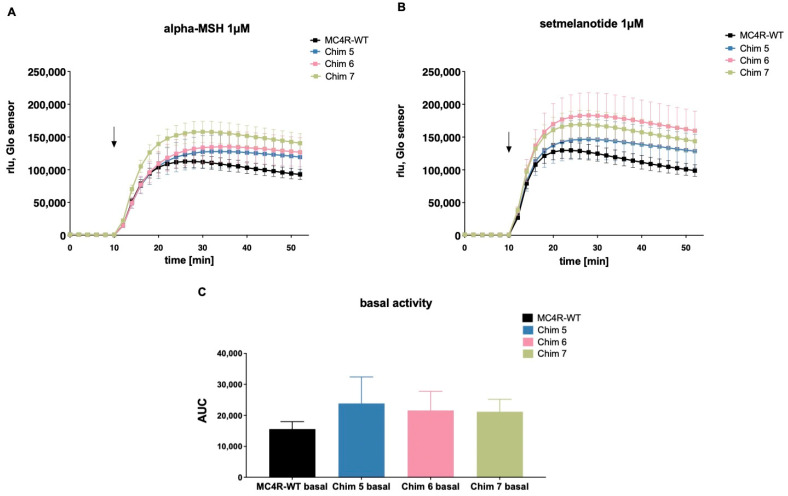
**G_s_ signaling properties of MC4R-WT and chimeric receptors under alpha-MSH and setmelanotide stimulation.** HEK293 cells were transfected with WT receptor or chimeric receptors and GloSensor™ reporter. Cells were stimulated with (**A**) alpha-MSH (1 µM) or (**B**) setmelanotide (1 µM) and assayed for increase in cAMP. The arrow indicates the start of ligand stimulation. cAMP accumulation was assessed over time and quantified in relative light units (rlu). (**C**) Chimeric receptors showed a higher basal G_s_ activity compared to MC4R-WT. The data are shown as area under the curve (AUC) of luminescence values from live cell cAMP accumulation. For statistical analysis, a one-way ANOVA with Kruskal–Wallis test was performed, comparing area under the curve of MC4R-WT to chimeric receptors stimulated with alpha-MSH or setmelanotide. The results turned out to be non-significant. Data represent four independent experiments, each performed in triplicate. Values represent mean ± SEM.

In a previous study, we presented data showing chimeric receptors exhibiting higher basal cAMP levels and higher NDP-alpha-MSH (a non-selective MCR agonist [23,28]) stimulated cAMP levels, compared to MC4R-WT [21]. Results were based on cAMP accumulation assays in COS-7 cells. In this study, we replicated the results for Chim 5, Chim 6, and Chim 7 in human HEK293 cells. Experiments were performed using GloSensor^TM^ assay enabling dynamic live cell measurement of cAMP increase. In order to compare the results of this study with results previously achieved in COS-7 cells, we used the same concentration of 1 µM for alpha-MSH and setmelanotide. In the current study, effects of setmelanotide stimulation on cAMP production of chimeric receptors were evaluated. GloSensor^TM^ results showed that alpha-MSH stimulation of Chim 5, Chim 6, and Chim 7 exhibited elevated cAMP production compared to WT (Figure 3A). A one-way ANOVA with Kruskal–Wallis test was performed, comparing area under the curve of MC4R-WT to Chim5, Chim 6, and Chim 7, stimulated with alpha-MSH or setmelanotide. The results turned out to be non-significant. The experiments were performed in triplicate of four independent experiments. This discrepancy might be explained by the different cell system and method used. Setmelanotide stimulation led to a similar increase in cAMP production in the chimeric receptors, compared to respectively alpha-MSH-induced cAMP increase (Figure 3B). Chim 5 to 7 exhibited qualitatively elevated basal cAMP levels compared to WT (Figure 3C).

### 3.3. Effects of Reduced Homodimerization on G_q/11_ Signaling

#### 3.3.1. Chimeric Receptors Exhibited Similar Basal Activity and Increased Efficacy as Well as Improved Potency Compared to MC4R-WT

**Figure 4:**
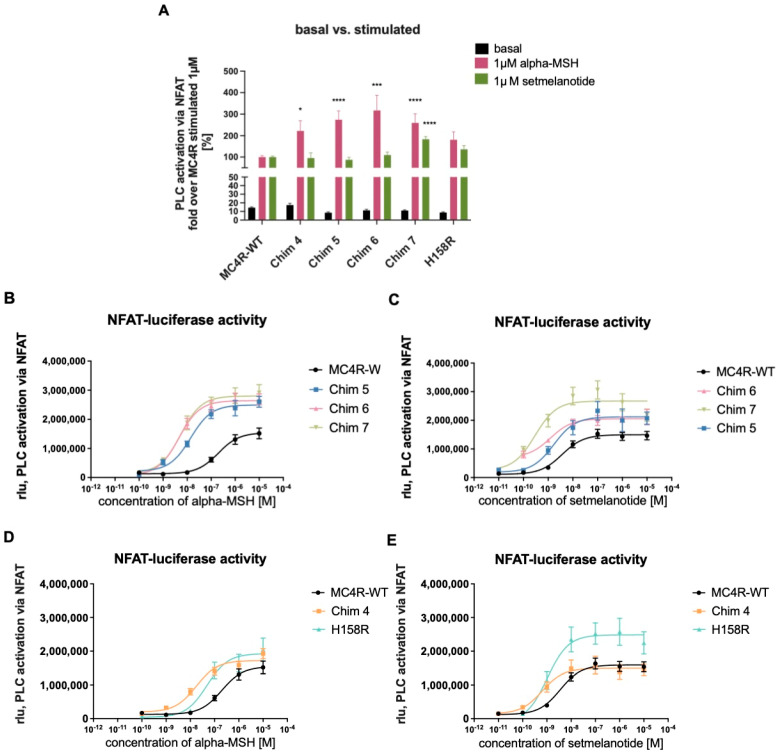
**G_q/11_ signaling of chimeric receptors and the H158R mutation compared to MC4R-WT.** NFAT-reporter gene assay quantifying relative light units (rlu) to determine G_q/11_ signaling capacities. HEK 293 cells were transfected with MC4R-WT, chimeric receptors, or H158R mutation. (**A**) Basal activity and 1 µM stimulated values of chimeric receptors and the H158R mutation compared to MC4R-WT. Basal activity of chimeric receptors is very similar to MC4R-WT. Chim 4, Chim 5, Chim 6, and Chim 7 displayed significant alpha-MSH induced increase in G_q/11_ activity compared to alpha-MSH stimulated MC4R-WT. Values represent fold over 1 µM stimulated MC4R-WT. For statistical analysis, a one-way ANOVA with Kruskal–Wallis test was performed, comparing MC4R-WT to chimeric receptors and the H158R mutation. Values represent mean ± SEM. Statistical significance is indicated by * *p* < 0.05, *** *p* < 0.001, and **** *p* < 0.0001. Concentration response curves of Chim5, Chim 6, and Chim 7 cells were stimulated with different concentrations of (**B**) alpha-MSH or (**C**) setmelanotide. Concentration response curves of Chim 4 and H158R mutation were stimulated with different concentrations of (**D**) alpha-MSH or (**E**) setmelanotide. Data are given as raw rlu. Data represent WT (alphaMSH n = 10, setmelanotide n = 17), H158R (n = 4), Chim 4 (alpha-MSH n = 5, setmelanotide n = 7), Chim 5 (n = 4), Chim 6 (alpha-MSH n = 4, setmelanotide n = 3), and Chim 7 (alpha-MSH n = 9, setmelanotide = 10) experiments, each performed in triplicate. Values represent mean ± SEM. E_max_ and EC_50_ values are summarized in Table 1.

Next, all chimeric receptors and the H158R mutation were tested for G_q/11_ signaling properties. For this purpose, we conducted reporter gene assays, measuring phospholipase C (PLC) activity through nuclear factor of activated T cell (NFAT) responsive element (Figure 4). All tested MC4R/CB1R chimeras showed similar basal G_q/11_ activity compared to MC4R-WT. Chim 4, Chim 5, Chim 6, and Chim 7 exhibited significantly elevated PLC activation via G_q/11_ after alpha-MSH stimulation (1 µM). The mutation H158R also exhibited higher efficacy (Emax) compared to MC4R-WT. Setmelanotide stimulation only led to a significant increase in efficacy for Chim 7, compared to MC4R-WT (Figure 4A). Table 1 shows the evaluation of chimeric receptor G_q/11_ signaling capacities compared to MC4R-WT after challenge with alpha-MSH or setmelanotide. The values are derived from concentration response experiments (Figure 4B–E). Reduced homodimerization results in a prominent effect on receptor potency. Intriguingly, EC_50_ values of Chim 6 (5.85 ± 1.29 nM) and of Chim 7 (6.33 ± 1.1 nM) after alpha-MSH stimulation were similar to EC_50_ of MC4R-WT after setmelanotide challenge (4.04 ± 0.5 nM). Of note, all chimeric receptors as well as the H158R mutation presented improved potency, compared to MC4R-WT EC_50_ value after alpha-MSH and after setmelanotide stimulation. Significant decrease in potency could be shown for Chim 4, Chim 6, and Chim 7 after alpha-MSH stimulation and for Chim 4 and Chim 7 after setmelanotide challenge, compared to MC4R-WT. The effects of impaired MC4R homodimerization on G_q/11_ signaling were also assessed through calculating the signaling bias. In particular, after alpha-MSH stimulation, all chimeric receptors and the H158R mutation showed a strong bias towards PLC activation compared to MC4R-WT. Chimeric receptors and H158R mutation also demonstrated a bias towards PLC activation after challenge with setmelanotide (Table 1). 

#### 3.3.2. Treatment with Pertussis Toxin (PTX) to Discriminate between G_q/11_ and G_i_βγ Activation of PLC

**Figure 5:**
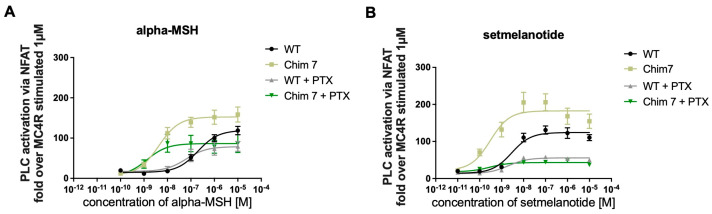
**NFAT reporter gene assay investigating signaling profile of PTX pretreated MC4R-WT-WT and Chim 7.** Calcium mobilization was measured using NFAT reporter gene assay. HEK293 cells were pretreated with PTX or not, assessing possible increase in Ca^2+^ due to G_i_ signaling effects. Data are given in percentage of MC4R-WT-WT signaling. Cells were stimulated with (**A**) alpha-MSH (fold over MC4R-WT at 1 µM was set as 100%) or with (**B**) setmelanotide (fold over MC4R-WT at 1 µM was set as 100%). E_max_ and EC_50_ values are summarized in Table 2. Data represent two to nine independent experiments, each performed in triplicate. Values represent mean ± SEM.

PLC activation can be a mixture of G_q/11_ activation and activation of βγ-subunits of G_i/0_ [29]. In order to determine whether the increase in PLC activation in chimeric receptors can fully be attributed to G_q/11_ activation, additional NFAT experiments were conducted including MC4R-WT and Chim 7. Cells were pretreated with pertussis toxin (PTX), a G_i/0_ inhibitor. In the presence of PTX, the G_i/0_ subunits are locked in their inactivate state, blocking this pathway [30]. Concentration–response curves after alpha-MSH challenge in the presence of PTX demonstrated a similar efficacy for MC4R-WT compared to signaling in the absence of PTX. For Chim 7, a reduction in efficacy compared to non-PTX treatment (significant) was observed. A shift towards lower EC_50_ values, indicating a significant decrease in potency for Chim 7 occurred, but not for MC4R-WT (Figure 5A, Table 2). After setmelanotide stimulation, the potency was only decreased for MC4R-WT but not for Chim 7 when comparing non-PTX to PTX data. As for efficacy, MC4R-WT as well as Chim 7 exhibited reduced maximal signaling via G_q/11_ in the case of G_i/0_ blockage through PTX (Figure 5B, Table 2). Focusing on the alpha-MSH, the natural endogenous ligand of MC4R, PTX treatment appeared to affect receptor efficacy more than it affected receptor potency. The blocking of G_i/0_ subunits led to a significant shift towards lower EC50 values for Chim 7 after alpha-MSH stimulation, indicating an increase in G_q/11_ signaling for Chim 7.

## 4. Discussion

The MC4R plays an essential role in weight regulation. Therefore, targeting the MC4R for anti-obesity treatment appeared obvious, but was rather unsuccessful for a long time [31]. The MC4R is a promiscuous receptor and able to activate different G proteins, thus presenting a broad signaling profile [25]. Subsequently, it is highly important to characterize the receptors’ full pharmacological profile and interaction capability. In this study, we analyzed effects of MC4R homodimerization on G_q/11_ subunit signaling, which appears to be a promising target regarding obesity treatment [12]. 

We recently showed dimer separation of the MC4R has a positive effect on G_s_ signaling. The study detected that multiple and single substitutions in the THM3-ICL2-TMH4 intracellular region of the MC4R inhibit dimerization, showing that these regions are important for homodimer formation. Hindering dimer formation led to an increase in basal and stimulated G_s_ signaling. These experiments were performed using COS-7 cells, and only G_s_ signaling was investigated [21]. 

These findings could be replicated in the present study using HEK293 cells and the GloSensor^TM^ technique, enabling live cell measurements. HEK293 cells are well established in GPCR research as they do not express the various MC receptors (MC1R-MC5R) [32]. COS-7 cells are derived from African green monkey (*Chlorocebus aethiops*) cell line [33], whereas HEK293 cells are derived from the human kidney cell line [34]. HEK 293 cell line is superior to COS-7 cells as this cell model allows for a closer approximation to human physiology, although in order to study MC4R function, it is still by far not optimal. One limitation of this model is that HEK293 cells cannot provide the same physiological setting that is present in the hypothalamic (*paraventricular nucleus)*, where most MC4R activity related to weight regulation is anticipated [10]. Additionally, the marginal presence of the melanocortin 2 receptor accessory protein 2 (MRAP2) in HEK293 cells, which possibly influences MC4R signaling and is discussed in detail below, should be taken into consideration [35,36]. 

Due to recent studies discussing the prominent role of the G_q/11_ activation, in terms of regulating energy homeostasis, it was compelling to investigate whether these findings also apply for MC4R dimer separation [7,26]. In this study, we showed that by inducing dimer separation, G_q/11_ subunit signaling was increased. Chim 6 and Chim 7 showed strongly decreased EC_50_ values after alpha-MSH stimulation compared to MC4R-WT potency. Setmelanotide was first approved in November 2020 by the U.S. FDA (USA) and is a novel medication for treating obesity caused by *POMC* and *LEPR* deficiency [37]. This cyclic peptide is a MC4R agonist that shows a strong bias towards G_q/11_ [7]. So far, study results showed that setmelanotide is not as effective in rescuing signaling in MC4R mutation patients, as it is in the treatment of *POMC* and *LEPR* deficiency [38]. Among other things, a reason for this might be that certain mutations can alter the receptor ligand binding domain, making it impossible for the ligand to bind to the mutated MC4R. Therefore, it was highly interesting to prove if MC4R dimer hindrance can have comparable effects on receptor potency compared to setmelanotide. Experimental results on the MC4R H158R mutation are in line with already existing data on the H158R, showing a bias towards G_q/11_ activation upon alpha-MSH stimulation [25]. We further provide data on the specificity of the H158R homodimer formation via NanoBRET^TM^, which has not been shown before (Figure S1).

In recent years, studies have revealed that GPCRs are expressed as a mixture of monomers and dimers, thus affecting the way how GPCR signal transduction occurs, e.g., [38,39]. New data identified that the expression pattern of monomer and dimer formation can be dynamic. For the corticotropin-releasing factor receptor type 1a, a monomer/dimer equilibrium was identified. These data suggest that other GPCRs might exist in a state of a monomer/dimer equilibrium [40,41]. Such data are currently not available for MC4R. Therefore, it would be of high importance to find out to what extent the MC4R exists in a dimeric state (homo- or hetero-dimeric states) to allow better characterization of the benefits that the induction of MC4R dimer separation might have on downstream signaling effects.

**Figure 6:**
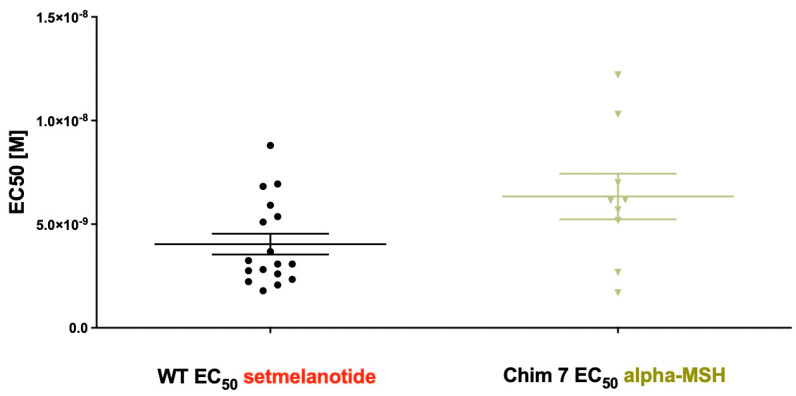
**EC_50_ of G_q/11_ signaling of MC4R-WT stimulated with setmelanotide compared to EC_50_ of Chim 7 stimulated with alpha-MSH**. Concentration–response curves were used to determine potency. EC_50_ of MC4R-WT after setmelanotide stimulation (4.04 ± 0.5 nM) appeared similar to EC_50_ of Chim 7 after alpha-MSH stimulation (6.33 ± 1.1nM). Data are given as the result of 9 to 17 independent experiments performed in triplicate. Values represent mean ± SEM.

It is striking that the induction of dimer separation had such a prominent effect on ligand-induced G_q/11_ signaling potency, as Chim 7 activated with alpha-MSH is comparable to MC4R-WT stimulated with setmelanotide (Figure 6). A possible explanation why dimer separation could lead to increased signaling capacities might be MC4R/G protein stoichiometry. In a dimeric state, only one G protein molecule can access the receptor dimer, due to steric reasons (stoichiometry 2:1). A monomerized receptor is able to couple one G protein molecule to each receptor (stoichiometry 2:2); this might cause higher signaling capacities due to doubled G protein activation [42]. This mechanism is important when wanting to discuss possibilities for therapeutical interventions. A protein of interest could be the accessory protein MRAP2. MRAP2 is hypothesized to interact with MC4R in particular by causing steric separation of the MC4R homodimers. Results showed that co-expression of MRAP2 with MC4R enhances G_s_ signaling. This makes MRAP2 an interesting endogenous allosteric factor when discussing dimer separation and its benefits [36].

Taken together, the study results provide information on the positive effect that inducing dimer separation of the MC4R has on G_s_ as well as G_q/11_ signaling capacities in human HEK293 cells. The results showed that dimer separation especially affects receptor potency, resulting in a shift towards lower EC_50_ values. Further investigation on MC4R monomer/dimer equilibrium is required to fully understand possible benefits of this approach. Future experiments ought to focus on finding ways to successfully induce dimer separation of the MC4R, perhaps allowing dimer separation to become a means of therapeutical intervention, helping a broader spectrum of obese patients.

## Figures and Tables

**Table 1:** **Efficacy (E_max_), potency (EC_50_), and bias of ligand stimulated MC4R-WT, chimeric receptors, and H158R.** Concentration–response curves were used to determine potency under alpha-MSH and setmelanotide stimulation. Data are given as the result of four to nine independent experiments performed in triplicate. Values represent mean ± SEM. Statistical analysis was performed using a one-way ANOVA with Kruskal–Wallis test comparing E_max_ of WT to variants or EC_50_ of WT to the variants. Statistical significance is indicated by a * *p* < 0.05, b ** *p* < 0.01, c *** *p* < 0.001, and d **** *p* < 0.0001. For calculation of bias, MC4R-WT serves as a reference and is plotted at a value of 1. The bias (chimeras versus MC4R) is stronger after alpha-MSH stimulation than after setmelanotide stimulation. The values are derived from EC_50_ and E_max_ values and originate from the concentration–response curves. Data are given as a result of four to nine independent experiments performed in triplicate. Data of relative dimerization compared to WT originates from Figure 2 NanoBRET^TM^ data.

MC4R-WT/ MC4R/CB1R Chimera	Alpha-MSH			Setmelanotide			NanoBRET^TM^
	E_max_ at 1 µM (Fold over MC4R-WT Stimulated 1 µMl)	EC_50_ (nM)	Bias	E_max_ at 1 µM (Fold over MC4R-WT Stimulated 1 µMl)	EC_50_ (nM)	Bias	Relative Dimerization Compared to WT
MC4R-WT	100	264 ± 65.9	1	100	4.04 ± 0.5	1	0
Chim 4	222.32 ± 47.50 a	14.7 ± 6.69 a	39.89	115.16 ± 15.10	0.71 ± 0.17 c	3.05	−32.55
Chim 5	274.42 ± 41.18 d	16.4 ± 6.06	44.22	87.31 ± 11.58	1.46 ± 0.38	6.58	−41.83
Chim 6	317.48 ± 70.5 c	5.85 ± 1.29 b	**143.14**	109.76 ± 13.20	1.25 ± 0.11	2.41	−15.19
Chim 7	259.59 ± 42.18 d	6.33 ± 1.1 c	**108.13**	182.83 ± 13.18 d	0.43 ± 0.12 d	17.32	−41.19
H158R	180.32 ± 37.67	49.2 ± 11.2	9.67	135.93 ± 17.16	1.8 ± 0.68	3.05	−28.94

**Table 2:** **Potency (EC_50_) of ligand stimulated MC4R-WT and Chim 7 including PTX pretreatment.** Concentration-response curves were used to determine potency under alpha-MSH and setmelanotide stimulation. Data are given as the result of two to nine independent experiments performed in triplicate. Values represent mean ± SEM. Statistical analysis was performed using the Mann–Whitney test comparing E_max_ of WT or Chim 7 to respective WT + PTX or Chim 7 + PTX. The same testing was performed for statistical EC_50_ analysis and comparison. Statistical significance is indicated by * *p* < 0.05, ** *p* < 0.01, and **** *p* < 0.0001.

MC4R-WT/Chim7	Alpha-MSH		Setmelanotide	
	E_max_ at 1 µM (Fold over MC4R-WT Stimulated 1 µMl)	EC_50_ (nM)	E_max_ at 1 µM (Fold over MC4R-WT Stimulated 1 µMl)	EC_50_ (nM)
MC4R-WT	100	264 ± 65.9	100	4.04 ± 0.5
MC4R-WT + PTX	96.98 ± 14.22	396 ± 313	70.26 ± 9.13 **	1.91 ± 0.36 *
Chim 7	259.59 ± 42.18	6.33 ± 1.1	182.83 ± 13.18	0.43 ± 0.12
Chim 7 + PTX	100.26 ± 13.8 **	1.8 ± 0.51 *	67.17 ± 12.40 ****	0.45 ± 0.08

## Data Availability

Data are contained within the article or Supplementary Material. The data presented in this study are available in Table S1 “Source data file”.

## Supplementary Materials

The following supporting information can be downloaded at https://www.mdpi.com/article/10.3390/biom12081119/s1, Figure S1: Donor saturation assays (DSA) investigating the specificity of receptor–receptor interaction of WT, Chim 7, and H158R.
