# Supplementary material for: A Setmelanotide-like Effect at MC4R Is Achieved by MC4R Dimer Separation
Source: Biomolecules. 2022 Aug 15;12(8):1119. doi: 10.3390/biom12081119 (PMC9405727; doi:10.3390/biom12081119)
Supplement: Supplementary file 1 [file biomolecules-12-01119-s001.zip › Supplements_final.pdf]

Supplements S1: Donor Saturation Assays (DSA) investigating the specificity of receptor-receptor interaction of WT, Chim7 and H158R.

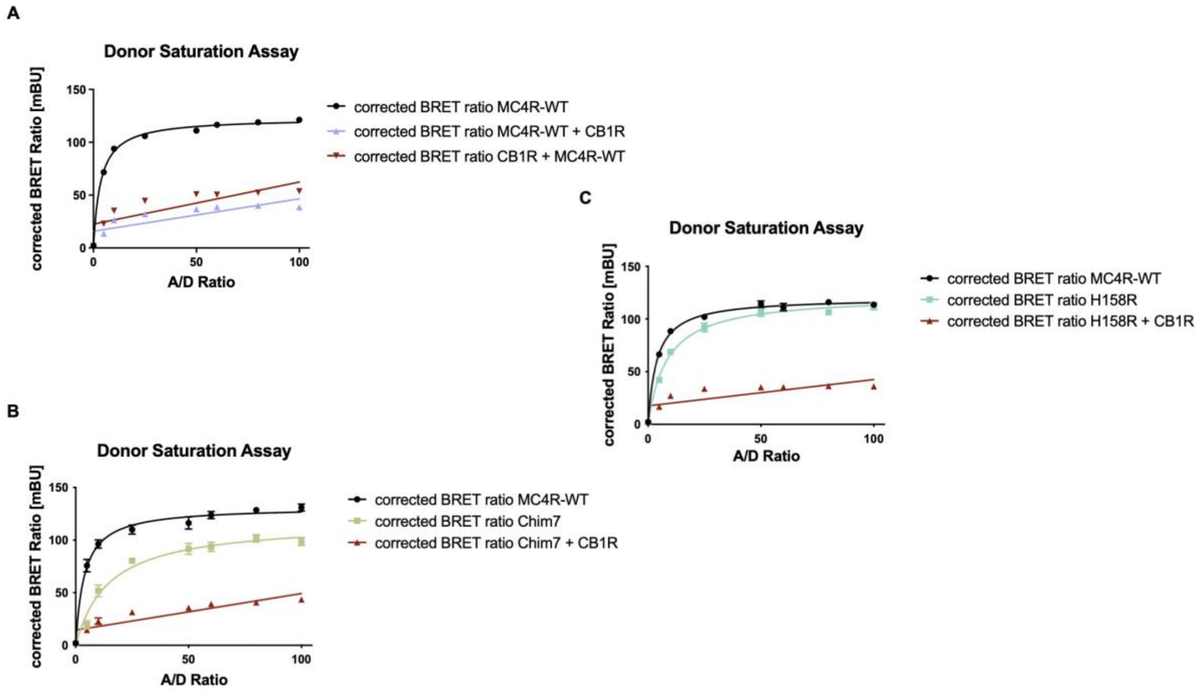

Figure S1: Donor Saturation Assays (DSA) investigating the specificity of receptor-receptor interaction of WT, Chim7 and H158R. HEK293 cells were transfected with increasing amounts of acceptor while the amount of donor was held constant. A receptor interaction is called specific, if a saturation level (B_{max}) can be achieved. This is represented by a hyperbolic curve. In a non-specific interaction a linear increase is expected. WT-MC4R served as positive control, CB1R was included as negative control as it is known not to interact with the MC4R. (A) Formation of WT-MC4R homodimers displays specific receptor-receptor interaction, represented by the black hyperbolic curve. The negative controls depict flat behavior. (B) Homodimerization of Chim 7 is depicted next to WT-MC4R homodimer formation. Interaction of Chim7 NL with CB1R HT served as negative control. Chim 7 shows specific dimerization in DSA. (C) Homodimerization of H158R is depicted next to positive and negative control. H158R shows specific homodimer formation. Data represent three independent experiments each performed in triplicates. Values represent mean ± SEM.
